# Clinical implications of microRNAs in human glioblastoma

**DOI:** 10.3389/fonc.2013.00019

**Published:** 2013-02-07

**Authors:** Masahiro Mizoguchi, Yanlei Guan, Koji Yoshimoto, Nobuhiro Hata, Toshiyuki Amano, Akira Nakamizo, Tomio Sasaki

**Affiliations:** Department of Neurosurgery, Graduate School of Medical Sciences, Kyushu UniversityFukuoka, Japan

**Keywords:** glioblastoma, temozolomide, microRNA, exosome

## Abstract

Glioblastoma (GBM) is one of the most common and dismal brain tumors in adults. Further elucidation of the molecular pathogenesis of GBM is mandatory to improve the overall survival of patients. A novel small non-coding RNA molecule, microRNA (miRNA), appears to represent one of the most attractive target molecules contributing to the pathogenesis of various types of tumors. Recent global analyses have revealed that several miRNAs are clinically implicated in GBM, with some reports indicating the association of miRNA dysregulation with acquired temozolomide (TMZ) resistance. More recent studies have revealed that miRNAs could play a role in cancer stem cell (CSC) properties, contributing to treatment resistance. In addition, greater impact might be expected from miRNA-targeted therapies based on tumor-derived exosomes that contain numerous functional miRNAs, which could be transferred between tumor cells and surrounding structures. Tumor-derived miRNAs are now considered to be a novel molecular mechanism promoting the progression of GBM. Establishment of miRNA-targeted therapies based on miRNA dysregulation of CSCs could provide effective therapeutic strategies for TMZ-resistant GBM. Recent progress has revealed that miRNAs are not only putative biological markers for diagnosis, but also one of the most promising targets for GBM treatment. Here in, we summarize the translational aspects of miRNAs in the diagnosis and treatment of GBM.

## INTRODUCTION

Glioblastoma (GBM) is one of the most common and malignant primary brain tumors in adults (Louis et al., 2007). The recent introduction of concomitant temozolomide (TMZ) with radiotherapy has led to prolonged overall survival of GBM patients. Furthermore, a small subgroup has shown favorable prognosis with survival exceeding 5 years (Stupp et al., 2009). However, median overall survival remains around 14.6 months, and the 5-year survival rate is only 9.8% at present (Stupp et al., 2009). In particular, a methylated O-6-methylguanine-DNA methyltransferase (MGMT) group showed prolongation of overall survival from 15.3 to 23.4 months using TMZ. Conversely, an unmethylated MGMT group showed no significant benefit with TMZ (11.8 vs. 12.6 months; Stupp et al., 2009). Further development of diagnosis and treatment based on novel molecular mechanisms is necessary for GBM patients, particularly for those with unmethylated MGMT.

**FIGURE 1:**
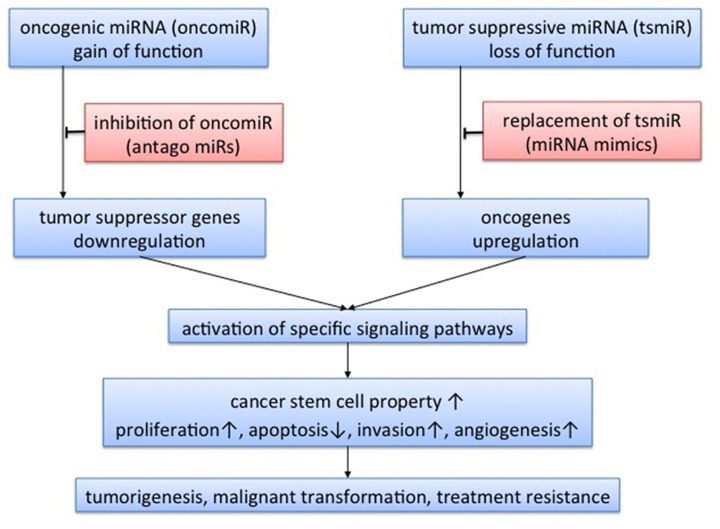
**Concept of microRNA-targeted therapy**. Expression of target genes is negatively regulated by microRNA (miRNA) via mRNA cleavage or translational inhibition. Therefore, miRNA acts as oncogenic miRNA when the target genes are tumor suppressor genes, and as tumor-suppressive miRNA when the target genes are oncogenes. Through regulation of tumor-related genes, miRNA could activate specific signaling pathways. A single miRNA could regulate expression of multiple genes, so the functions of miRNA are varied, affecting cancer stem cell properties, tumor proliferation, apoptosis, invasion, and angiogenesis. miRNA has the potential to play roles in tumorigenesis, malignant transformation, and treatment resistance. Theoretically, there are two approaches to miRNA-targeted therapy: inhibition of oncogenic miRNA with antago-miRs; and replacement of tumor-suppressive miRNA with miRNA mimics.

A class of small non-coding RNA molecules, named microRNA (miRNA), was recently identified and revealed to regulate a wide spectrum of gene expression in a post-transcriptional manner (Ambros, 2004; Bartel, 2004). More than 1500 precursors and 1921 mature *Homo sapiens* miRNAs have been identified and registered in miRBase to date (Kozomara and Griffiths-Jones, 2011). Primary miRNA (pri-miRNA) is transcribed in the nucleus, and spliced to precursor miRNA (pre-miRNA) by Drosha complexes. Pre-miRNA is then transferred to the cytoplasm by exportin-5, followed by further processing for mature miRNA by Dicer complexes. Mature miRNA is incorporated into an effector complex known as an RNA-induced silencing complex (RISC), which binds to RNA (mRNA) and can affect the translation and stability of mRNA (Filipowicz et al., 2008). Expression of the target mRNA is regulated, either by mRNA cleavage or by translational inhibition, depending on the complementarity of “seed” sequences (Filipowicz et al., 2008; Bartel, 2009). Recent reports have revealed that miRNAs play crucial roles in tumorigenesis, angiogenesis, invasion, and apoptosis in various types of tumor (Ambros, 2004; Bartel, 2004; **Figure 1**). In addition, miRNA expression profiling may yield more accurate classifications of human cancers than mRNA expression profiling (Lu et al., 2005). The present review summarizes the clinical implications of miRNA in GBM and future directions of miRNA-based therapy for GBM.

## IDENTIFICATION OF GLIOMA-RELATED miRNA

Calin (2002) provided the first report of the frequent deletions and down-regulation of miR-15 and miR-16 at chromosome 13q14 in a majority of cases of chronic lymphocytic leukemia, and also demonstrated that more than half of miRNA genes are located in cancer-associated genomic regions or in fragile sites (Calin, 2004). In addition, Lu et al. (2005) reported that the classification of malignant tumors based on miRNA expression profiles was more precise than that based on mRNA expression profiles. These findings have added impetus to miRNA research in the field of oncology. In 2005, two reports identified aberrant miRNA expression in GBM using microarray analysis (Chan et al., 2005; Ciafre et al., 2005). A common miRNA identified by both groups, miR-21, has been revealed to act as an anti-apoptotic factor (Chan et al., 2005; Papagiannakopoulos et al., 2008). Indeed, miR-21 represents a unique miRNA overexpressed in almost all types of tumor investigated to date. In addition, a recent *in vivo* study revealed that miR-21 plays a critical role in various steps of tumor progression (Medina et al., 2010). This study also proved for the first time that certain tumors addict to specific miRNAs. The concept of “oncomiR addiction,” similar to the phenomenon of “oncogenic addiction,” would also facilitate study of the therapeutic application of miRNAs in human cancer. Two studies using microarray analysis subsequently identified miR-128 and miR-10b, and investigated the biological function of these miRNAs (Godlewski et al., 2008; Sasayama et al., 2009). The stem-loop reverse transcription-polymerase chain reaction (RT-PCR) array, which can evaluate miRNA expression with superior sensitivity and specificity to microarrays, has been applied to investigations using miRNA profiling (Chen et al., 2009; Mestdagh et al., 2009). Our previous study using the array identified 16 miRNAs for which expression was significantly altered in GBM (WHO grade 4) compared with anaplastic astrocytoma (WHO grade 3), and demonstrated that overexpression of miR-196a/b correlates with shorter overall survival (Guan et al., 2010). Malzkorn et al. (2010) also identified 12 upregulated miRNAs involved in the malignant progression of gliomas using stem-loop real-time RT-PCR. Kim et al. (2010) also reported clinical implications of miR-26 gene amplification in GBM patients using TCGA data. Rao et al. (2010) recently used a locked nucleic acid array to generate a large-scale, genome-wide miRNA expression profile, and identified 55 upregulated and 29 downregulated miRNAs in malignant gliomas. A more important finding in the study was that a cluster of only 23 miRNAs was sufficient to distinguish GBM from anaplastic astrocytoma with 95% accuracy (Rao et al., 2010). Furthermore, Srinivasan et al. (2011) identified a 10-miRNA expression signature that could predict overall survival of GBM patients. More recently, a combined mRNA and miRNA expression profiling signature has been used to identify five GBM subclasses (Kim et al., 2011).

Dysregulated miRNAs, as identified by recent global studies for GBM, were summarized in our previous review (Mizoguchi et al., 2012). Each report has demonstrated several miRNAs for which expression was significantly altered in GBM compared with normal brain tissues or lower-grade gliomas. Taken together, 52 upregulated miRNAs and 33 downregulated miRNAs have been reported in seven global studies published between 2005 and 2010 (Mizoguchi et al., 2012). The results of each report were quite varied. One of the causes of such discrepancies is differences in methodology, platform, and control samples. Recurrent aberrations of expression were thus detected in only four miRNAs (miR-21, miR-10b, miR-128-1, and miR128-2). In fact, recently identified miRNAs have not been included in previous studies, but the number of concordances among these studies is extremely small. It is obvious, however, that these four miRNAs have the potential to contribute to the molecular pathogenesis of GBM (**Table 1**).

**Table 1:** Candidate targets of miRNA in glioblastoma.

miR-21	Most common oncomiR in a wide range of cancers, acting as an anti-apoptotic factor targeting a network of p53, transforming growth factor beta (TGF-β), and mitochondrial apoptosis tumor suppressor genes
miR-10b	Commonly upregulated miRNA in glioblastoma, located in HOX cluster
miR-128	miRNA associated with glioma stem cell properties and neuronal differentiation via Bmi-1 and epidermal growth factor receptor (EGFR)/platelet-derived growth factor (PDGF)/AKT signaling pathways
miR-34b	One of the most elucidated tumor suppressor miRNAs, considered a key regulator of tumor suppressor pathways; one of the promising targets for miRNA replacement therapy
miR-196	Extremely highly expressed miRNA in glioblastoma showing significant association with overall survival

## SUBCLASSIFICATION BASED ON miRNA EXPRESSION PROFILES

Based on molecular pathological perspectives, GBM is a heterogeneous tumor. To elucidate novel therapeutic approaches, therefore, more precise stratification is necessary. Recent integrated genomic analyses have allowed molecular subclassification of GBM based on gene expression profiles (Phillips et al., 2006; Verhaak et al., 2010; Huse et al., 2011). Two recent classifications based on mRNA expression profiles have been reported, with both identifying one of the subclasses as showing an expression signature resembling that of a “proneural” precursor cell (Phillips et al., 2006; Verhaak et al., 2010). However, the other subclasses of GBM have shown little association with neural differentiation, and disagreement remains regarding the relationship of subclasses to clinical outcomes (Phillips et al., 2006; Verhaak et al., 2010). Establishment of more efficient diagnostic and therapeutic strategies based on molecular stratification is a focus of current research.

Recent attempts at miRNA expression profiling may indicate the way toward more accurate classification of human cancers than mRNA expression profiling (Lu et al., 2005). Kim et al. (2011) identified five clusters of GBM based on miRNA expression profiles, which appeared to predict clinical outcomes more precisely than mRNA profile. Expression profiles of miRNA could lead to the identification of novel therapeutic targets based on clinically useful subclassifications of GBM. Recent reports, however, have identified significant associations between miRNA and mRNA expression signatures (Wuchty et al., 2011; Ma et al., 2012b). Further clarification of the physical interactions between miRNA and mRNA is necessary for more precise and practical stratification of GBM.

## PROGNOSTIC VALUE OF miRNA EXPRESSION PROFILES

We recently revealed that expressions of miR-196a and miR-196b are extremely high compared with other overexpressed miRNAs in GBM (Guan et al., 2010). Furthermore, we identified a significant association between high expression of miR-196a/b and shorter overall survival among GBM patients, so both of these miRNAs are considered to be associated with the malignant transformation of gliomas (Guan et al., 2010). Overexpression of miR-196 has also been identified in several other cancers (Gaur et al., 2007), and Bloomston et al. (2007) reported a correlation between miR-196a-2 and shorter overall survival in patients with pancreatic cancer. Another recent report validated the prognostic value of miR-196b in a large cohort of GBM (Ma et al., 2012a). As described above, Kim et al. (2011) also identified five clusters of GBM based on miRNA expression profiles, predicting clinical outcomes more precisely than mRNA profiles. In addition, a 10-miRNA signature was identified to predict survival of GBM patients using TCGA data (Srinivasan et al., 2011). Expression profiles of miRNA are useful for predicting GBM patient survival, and have the potential to identify efficacious therapeutic targets.

## miRNA EXPRESSION ASSOCIATED WITH TMZ RESISTANCE

Since the introduction of TMZ as standard chemotherapy, concomitant TMZ and radiotherapy have improved both progression-free and overall survival in patients with GBM (Stupp et al., 2005). However, the clinical prognosis of patients with GBM remains poor, with a median overall survival of only 14.6 months (Stupp et al., 2005). Furthermore, molecular-targeted therapy for single genes could not achieve prolonged overall survival of GBM patients (Weller et al., 2012). Further molecular mechanisms underlying treatment resistance need to be elucidated for malignant gliomas, and miRNA offers an attractive molecular target to improve the effects of conventional therapies.

In fact, recent miRNA studies have revealed that aberrant miRNA expression could affect chemosensitivity, and have also identified several miRNAs (i.e., miR-21, miR-125b-2, miR-195, miR-455-3p, miR-10a) associated with TMZ resistance (Shi et al., 2010; Ujifuku et al., 2010; Zhang et al., 2012). Recent reports have demonstrated that TMZ-induced apoptosis is inhibited by miR-21 overexpression by decreasing the Bax/Bcl-2 ratio and caspase-3 activity in GBM cell lines (Shi et al., 2010; Zhang et al., 2012). Ujifuku et al. (2010) also identified three miRNAs (miR-195, miR-455-3p, and miR-10a*) overexpressed in the induced TMZ-resistant cell line, and demonstrated that miR-195 inhibition enhanced TMZ-induced cell death. All of these reports indicated that miRNAs play a role in TMZ resistance, and regulation of the specific miRNA combined with TMZ could enhance TMZ-induced cell death. As described above, specific miRNAs are involved in critical signaling pathways of GBM (Sana et al., 2011), so the regulation of aberrant miRNA expression could affect not only conventional radio- and chemosensitivity, but also sensitivities to molecular-targeted therapy. The combination of miRNA-targeted therapy and conventional chemotherapy or molecular-targeted therapy has the potential to enhance treatment efficacy, particularly for patients with TMZ-resistant GBM.

## miRNA EXPRESSION ASSOCIATED WITH RADIORESISTANCE

Radiotherapy is another effective cytotoxic therapy for GBM patients. In 2007, Weidhaas et al. (2007) first demonstrated the association of overexpression of the let-7 family of miRNAs and radiosensitive state *in vitro* in lung cancer cells and *in vivo* in *C. elegans*. Thereafter several reports revealed that miRNA dysregulation also affect the radiosensitivity of glioma cells (Chen et al., 2010; Lee et al., 2011; Li et al., 2011; Gwak et al., 2012). In addition, global gene expression profile revealed that ionizing radiation induced significant alterations of miRNA expression in tumor cells (Niemoeller et al., 2011). Several miRNAs are associated with DNA double-strand breaks (DSBs) repair system including non-homologous end joining (NHEJ) and homologous recombination (HR). For instance, a catalytic subunit of DNA-dependent protein kinase (DNA-PKs) is inhibited by miR-7 and miR-101 (Yan et al., 2010; Lee et al., 2011), and ataxia telangiectasia mutated protein (ATM) is regulated with miR-18a, miR-100, miR-101, and miR-421 (Ng et al., 2010; Yan et al., 2010). The regulation of DSBs repair system by miRNA replacement could provide a novel way to increase vulnerability to radiotherapy. MiR-21, commonly upregulated in GBM, also play a crucial role for radiosensitization of GBM by modulating a tumor suppressor network and phosphoinositide 3-kinase (PI3K)/AKT pathway (Papagiannakopoulos et al., 2008; Gwak et al., 2012). Further evaluation of miRNA associated with cancer stem cell (CSC) properties is also required, because CSCs contribute enhanced radioresistance due to greater activation of DNA-repair responses (Bao et al., 2006).

## miRNA AND CANCER STEM CELL PROPERTIES

The CSC hypothesis, that tumors are driven by a small subpopulation of tumor cells with stem cell-like properties, may provide novel insights into the radio- and chemoresistance of GBM (Magee et al., 2012). Based on this hypothesis, some therapies in which tumors show reduced diameter might not be associated with improved rates of cure if CSCs fail to be eliminated, and therapies targeting CSCs should thus prove clinically more effective (Reya et al., 2001; Bao et al., 2006). Elucidating the molecular biology of CSCs thus appears crucial for resolving the mechanisms underlying treatment resistance.

Recently, some miRNAs have been reported to contribute to CSC properties and cancer heterogeneity. Godlewski et al. (2008) identified down-regulation of miR-128 in GBM compared with adjacent brain, leading to reduced self-renewal of glioma stem cells via Bmi-1 down-regulation. That report represented the first demonstration of an association between miRNA and stem cell properties in glioma. In addition to miR-128, miR-124, miR-137 (Silber et al., 2008), miR-34a (Li et al., 2009; Guessous et al., 2010), and miR-326 (Kefas et al., 2009) reportedly play roles in the maintenance of CSC properties. The identification of miRNAs thus adds another mechanism of gene expression associated with gliomagenesis and maintenance of CSC properties via the regulation of targeted signaling pathways (**Figure 1**).

## NOVEL TREATMENT STRATEGY BASED ON miRNA SIGNATURE

Dysregulation of miRNA expression has the potential to affect not only conventional radio- and chemotherapy, but also sensitivity to molecular-targeted therapy. Further study of miRNA function in CSCs may lead to novel treatment strategies for GBM. Consideration of the therapeutic application of miRNAs suggests two putative options: inhibition of oncogenic miRNAs; and replacement of tumor-suppressive miRNAs (**Figure 1**). Application of an antagonist of specific miRNAs (oncomiR), which would function as an oncogene, is one way of inhibiting oncogenic signaling pathways. Another is replacement therapy with mimics of miRNAs, to act as tumor suppressors. For both approaches, better delivery systems to target tissue remain under investigation. Multiple steps remain to achieve the targeted miRNA-based therapy. Inhibition of oncogenic molecules has been considered a more theoretically feasible approach. Considering replacement therapy, however, miRNA-based therapy could be more practical than conventional gene therapy, since miRNA is substantially smaller and more stable than protein-coding genes. Further elucidation of tumor suppressor miRNA thus has the potential to yield opportunities to develop replacement molecular therapies, which have not previously been considered as a realistic approach. In fact, recent research has facilitated the replacement of miRNAs for tumor suppressor miR-7, miR-128, and miR-34a over the inhibitory approach (Godlewski et al., 2008; Kefas et al., 2008; Bader, 2012). As a well-elucidated tumor suppressor miRNA for various types of tumors, miR-34a is now one of the most promising targets of miRNA replacement therapy (Bader, 2012). miR-34a, which is induced by p53, is considered to be a key regulator of tumor-suppressive signaling pathways in various cancers (Bader, 2012). Furthermore, recent reports have revealed that miR-34a plays a crucial role in the molecular pathogenesis of GBM (Li et al., 2009), although the function is more complex in GBM and remains under investigation (Genovese et al., 2012; Silber et al., 2012; Yin et al., 2012). Replacement therapy of miR-34a is an attractive approach for various malignant tumors, but should be introduced carefully for GBM.

Many obstacles must still be overcome to establish miRNA-targeted therapies. One of the most critical issues is how to deliver the agent (an miRNA mimic or inhibitor) to brains protected by the blood–brain barrier. Local delivery techniques could potentially improve the efficacy for GBM, since systematic metastasis is extremely rare in GBM compared with other types of tumor. Convection-enhanced delivery (CED) is one of the most promising local delivery techniques to improve the efficacy of recent treatments, including conventional chemotherapy, molecular-targeted therapy, and gene therapy (Zhou et al., 2012). For further improvement of CED, a nanoparticle delivery system should be introduced for the treatment of brain tumors (Hadjipanayis et al., 2010). These approaches would also provide the chance to create new delivery strategies for miRNA-based therapy.

Tumor-derived exosomes and secreted miRNA are novel and exciting targets for miRNA-based therapy. Recent investigations have revealed that exosomes secreted by tumor cells contain numerous functional miRNAs (Valadi et al., 2007), which could play important roles in tumor initiation and progression. Secreted miRNAs derived from tumor cells may play important roles in intercellular communication, since miRNA transferred from tumor cells could regulate protein expression in the surrounding structure (Katakowski et al., 2010). This intercellular mechanism represents a novel therapeutic target for the future, and a recent investigation into the biological function of exosomes suggests the opportunity to create a new miRNA delivery system (Alvarez-Erviti et al., 2011).

## CONCLUSION

As a novel molecule, miRNA may contribute to TMZ resistance in GBM and could offer a crucial biomarker not only as a diagnostic marker, but also as a target for molecular therapies. Therapies based on miRNA could achieve modest changes for more global gene expression, and may prove more effective than current molecular-targeted therapies, which target single protein-coding genes within an oncogenic signaling pathway. Molecular-targeted therapy based on miRNA expression profiles in CSCs has the potential to allow development of more radical treatment strategies in the future. Further investigation of miRNA biology in GBM could provide novel insights for the development of efficient treatment strategies, especially for TMZ-resistant GBM. Furthermore, investigations into tumor-derived exosomes are providing some of the most promising approaches for creating novel strategies for the diagnosis and treatment of GBM.

## Conflict of Interest Statement

The authors declare that the research was conducted in the absence of any commercial or financial relationships that could be construed as a potential conflict of interest.
